# Solubility, Solution Thermodynamics, and Preferential Solvation of Amygdalin in Ethanol + Water Solvent Mixtures

**DOI:** 10.3390/ph13110395

**Published:** 2020-11-16

**Authors:** Abdelkarim Aydi, Cherifa Ayadi, Kaouther Ghachem, Abdulaal Z. Al-Khazaal, Daniel R. Delgado, Mohammad Alnaief, Lioua Kolsi

**Affiliations:** 1 LETIAM, Lip (Sys)2, IUT d’Orsay, Université Paris-Sud, Plateau de Moulon, 91400 Orsay, France; 2 Laboratory of Materials Molecules and Applications, Preparatory Institute for Scientific and Technical Studies, University of Carthage, Tunis 1082, Tunisia; 3 Laboratory of Materials, Treatment and Analysis (LMTA), National Institute of Research and Physicochemical Analysis (INRAP), BiotechPole Sidi-Thabet, Ariana 2020, Tunisia; sherifa.ayadi@gmail.com; 4 Faculté des Sciences de Tunis, Université Tunis El Manar, Campus Universitaire El Manar, Tunis 2092, Tunisia; 5 Department of Industrial Engineering and Systems, College of Engineering, Princess Nourah bint Abdulrahman University, Riyadh 84428, Saudi Arabia; kgmaatki@pnu.edu.sa; 6 Department of Chemical and Materials Engineering, Faculty of Engineering, Northern Border University, Arar P.O. Box 1321, Saudi Arabia; abdulaal.alkhazaal@gmail.com; 7 GRIAUCC Research Group, Department of Engineering, Industrial Engineering Program, Universidad Cooperativa de Colombia, Calle 11 No. 1-51, Neiva 410001, Huila, Colombia; danielr.delgado@campusucc.edu.co; 8 Department of Pharmaceutical and Chemical Engineering, Faculty of Applied Medical Sciences, German Jordanian University, Amman 11180, Jordan; mohammad.alnaief@gju.edu.jo; 9 Department of Mechanical Engineering, College of Engineering, Ha’il University, Ha’il City 81481, Saudi Arabia; l.kolsi@uoh.edu.sa; 10 Laboratory of Metrology and Energy Systems, National Engineering School of Monastir, University of Monastir, Monastir 5000, Tunisia

**Keywords:** amygdalin, thermodynamics, van’t Hoff, Gibbs equation, solubility, Jouyban–Acree, Buchowski–Ksiazczak, inverse Kirkwood–Buff integral

## Abstract

The equilibrium solubility of amygdalin in [ethanol (1) + water (2)] mixtures at 293.15 K to 328.15 K was reported. The thermodynamic properties (standard enthalpy Δ_soln_H°, standard entropy Δ_soln_S°, and standard Gibbs energy of solution Δ_soln_G°) were computed using the generated solubility data via van’t Hoff and Gibbs equations. The dissolution process of amygdalin is endothermic and the driving mechanism in all mixtures is entropy. Maximal solubility was achieved in 0.4 mole fraction of ethanol at 328.15 K and the minimal one in neat ethanol at 293.15 K. Van’t Hoff, Jouyban–Acree–van’t Hoff, and Buchowski–Ksiazczak models were used to simulate the obtained solubility data. The calculated solubilities deviate reasonably from experimental data. Preferential solvation parameters of amygdalin in mixture solvents were analyzed using the inverse Kirkwood–Buff integrals (IKBI) method. Amygdalin is preferentially solvated by water in ethanol-rich mixtures, whereas in water-rich mixtures, there is no clear evidence that determines which of water or ethanol solvents would be most likely to solvate the molecule.

## 1. Introduction

**Figure 1:**
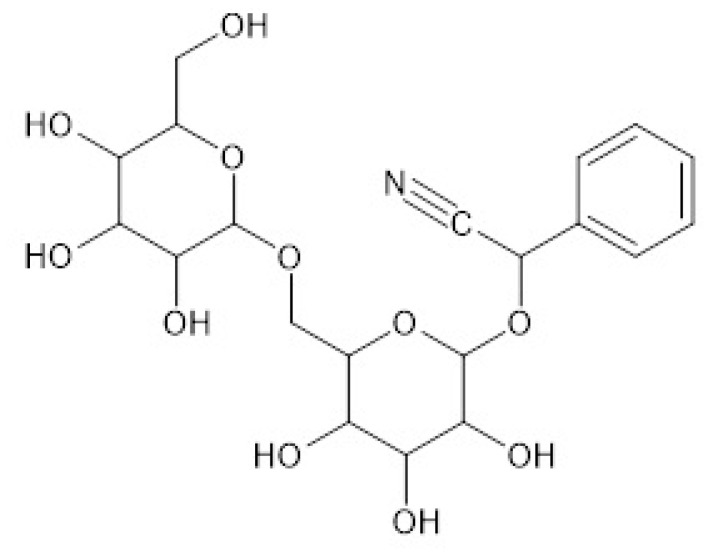
Molecular structure of amygdalin.

Amygdalin (Figure 1) is a naturally occurring cyanogenic diglycoside with a molecular formula of C_20_H_27_NO_11_ and a molecular mass of 457.4 g mol^−1^. It is a major bioactive component present mostly in kernels and seeds of “Rosaceae” plants such as peaches, apples, cherries, and more [1,2]. The use of amygdalin can lead to the release of toxic hydrogen cyanide (HCN) through the action of emulsin enzyme from the human intestinal microflora [3]. The HCN selectively decomposes cancer cells in the tumor site inside the body [4,5]. Several studies have demonstrated the antitumor activities of amygdalin on prostate cancer, bladder cancer, lung cancer, rectal cancer, and colon cancer [6]. Furthermore, highly purified amygdalin used in therapeutic dosage levels has antioxidant, anti-fibrosis [7], anti-inflammatory, analgesic [8,9], anti-atherosclerosis [10,11,12], anti-cardiac hypertrophy [13], anti-ulcer [14], anti-tussive, and anti-asthmatic effects [15].

Besides the degradation of amygdalin caused by enzymes from the gut microflora, plant enzymes (β-glucosidases and α-hydroxynitrile lyases) can lead to the production of cyanide when plant tissue is damaged or seeds are crushed or macerated. Enzymatic degradation of amygdalin to gentibiose, benzaldehyde, and HCN usually takes place in an alkaline solution [16].

In addition to enzymatic hydrolysis mentioned above, amygdalin degradation can also occur in boiling water through the process of epimerization, particularly under mild basic conditions as well as in a long extraction time [17,18,19]. In Bolarinwa et al.’s research [17], it was demonstrated that at 100 °C of boiling water, an extended extraction period can result in reduced extraction yield due to the conversion of amygdalin into neoamygdalin (amygdalin epimer).

Extracting a high rate of amygdalin from food plants without causing degradation of the molecule is challenging to achieve. Therefore, the selectivity of a solvent for this compound is a crucial parameter, as this will have a paramount influence on the extraction process. Extraction rate and time can be considerably affected by amygdalin solubility in solvents [17]. It is therefore of some interest to know the solubility of amygdalin in different mixtures of solvents. On the other hand, the knowledge of the solubility behavior in different solvent systems is of high importance in the pharmaceutical industry as it influences the drug efficacy and its pharmacokinetics [20]. Solubility data are also useful for drug purification, refining procedures, and method development [21,22,23].

For the above-mentioned reasons, the solubility and solution thermodynamics of amygdalin in pure and solvent mixtures are quite essential and must be determined. The binary water and ethanol mixtures are the most versatile and most used solvent systems for these previous purposes [24,25,26,27].

Thus, the goals of this study were (1) to extend the database on the solubility of amygdalin in several ethanol (1) + water (2) mixtures over a temperature range of 298.105 to 328.15 K, (2) to study the effect of solvent composition on the solubility and solution thermodynamics of amygdalin in aqueous ethanol mixtures, (3) to calculate the apparent thermodynamic functions of solution in the investigated solvents using the van’t Hoff and Gibbs equations, and (4) to estimate the preferential solvation of amygdalin in these solvents through the method of inverse Kirkwood–Buff integrals (IKBI), which describes the local solvent proportions around the dissolved substance concerning to the composition of the cosolvent mixtures [28,29]. Some models were used to predict the solubility of amygdalin in ethanol–water mixtures at different temperatures.

## 2. Results and Discussion

### 2.1. Solubility of Amygdalin in [Ethanol (1) + Water (2)] Cosolvent Mixtures

Table 1 shows the experimental solubility of amygdalin (3) in {ethanol (1) + water (2)} cosolvent mixtures including EtOH and water neat solvents at nine temperatures (293.15–328.15 K).

**Figure 2:**
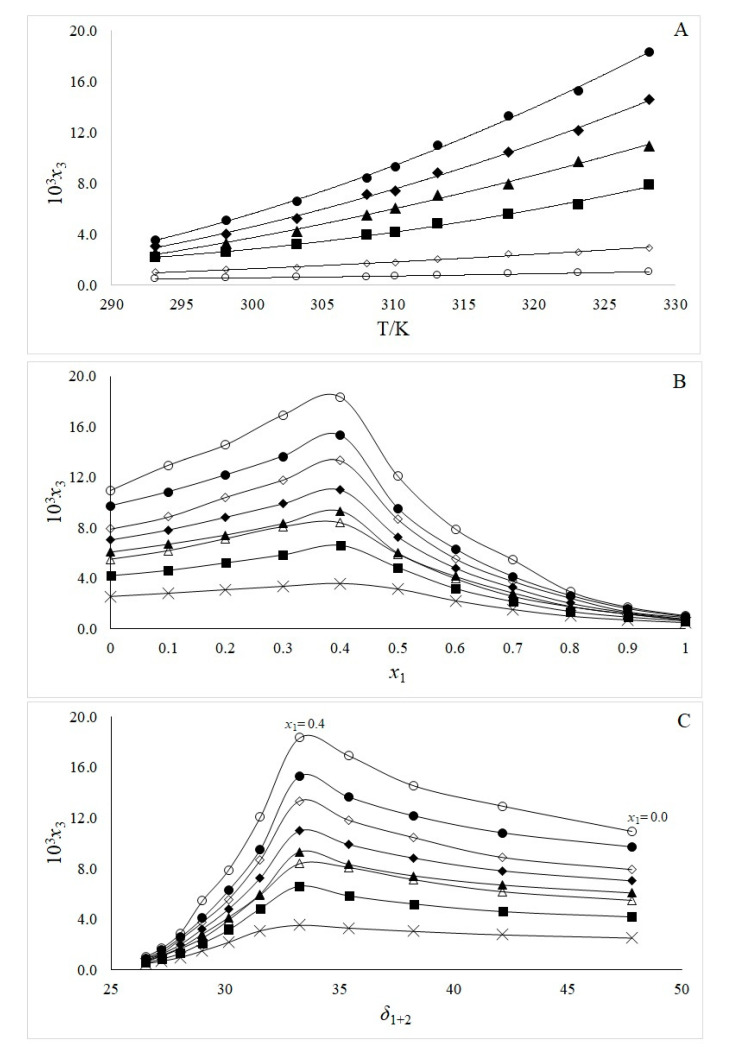
Experimental solubility of amygdalin (3) in mole fraction (10^3^x_3_) as a function of temperature (**A**); as a function of composition of solvent mixtures (**B**); and as a function of Hildebrand solubility parameter *δ*_1__+ 2_ of the [ethanol (1) + water (2)] mixtures (**C**). For A, ○: EtOH; ◊: x_1_ = 0.8; ■: x_1_ = 0.6; ▲: water; ♦: x_1_ = 0.2; and ●: x_1_ = 0.4, and for B and C, x: 293.15 K; □: 298.15 K; ■: 303.15; ∆: 308.15; ▲: 310.15 K; ♦: 313.15 K; ◊: 318.15 K; ●: 323.15 K; and ○: 328.15 K.

Experimental results demonstrate that the solubility increases with temperature indicating endothermic dissolution (Figure 2A). The maximum solubility of amygdalin was observed in the cosolvent mixture *x*_1_ = 0.4 at 328.15 K (Figure 2B). The addition of ethanol (in water-rich mixtures) has a positive cosolvent effect, enhancing amygdalin solubility. Indeed, the presence of the non-polar phenyl group in the amygdalin chemical structure may cause the formation of a structured water layer around it. As the proportion of ethanol in the solvent mixture increases, the solvation water shell will be ruptured [30,31,32], therefore, increasing amygdalin’s solubility in the system.

The solubility profiles of amygdalin in {ethanol (1) + water (2)} binary mixtures at different temperatures were plotted as a function of the Hildebrand solubility parameter *δ*_1 + 2_ of the mixtures (Figure 2C).

For binary mixtures, *δ*_1 + 2_ is calculated as [33]:

(1) $$\delta_{1\;+\;2}=f_{1}\delta_{1}+\left(1-f_{1}\right)\delta_{2}$$

where *δ*_1_ and *δ*_2_ are the Hildebrand solubility parameters of the pure solvents (*δ*_1_ = 26.5 MPa^1/2^ for ethanol (1) [34] and *δ*_2_ = 47.8 MPa^1/2^ for water (2) [34]; *f* is the solute-free volume fraction which is calculated assuming additive volumes as:

*f* = *V*_1_/(*V*_1_ + *V*_2_) (2)

where *V*_1_ and *V*_2_ are the volumes of cosolvent and water, respectively.

Considering the entire polarity region, the solubility increases from pure water (*δ* = 47.8 MPa^1/2^) up to the mixture with x_1_ = 0.40 (*δ*_mix_ = 33.2 MPa^1/2^), where the curve shows a maximum solubility peak; from this mixture up to pure ethanol, the solubility decreases in all cases (Figure 2C).

This behavior is commonly observed in compounds whose polarity coincides with the polarity of a mixture of solvents (*δ*_1_ > *δ*_3_ > *δ*_2_ or *δ*_2_ > *δ*_3_ > *δ*_1_) [35,36].

According to the literature, solutes reach their maximum solubility in solvents with the same solubility parameter [36] and thus, the *δ*_3_ value of amygdalin (3) would be 33.2 MPa^1/2^. However, the solubility parameter of amygdalin (3), estimated in accordance with the group contribution methods proposed by Fedors and van Krevelen, is *δ*_3_ = 29.9 MPa^1/2^ (Table 2), which is lower than the experimental value obtained in this work at the solubility maximum (*δ*_3_ = 33.2 MPa^1/2^).

It is important to note that the group contribution methods only provide a rough estimation of *δ*_3_; however, this calculation is relevant to identify the most suitable solvent or solvent mixture to dissolve the drug, which is useful information in experimental and industrial designs.

### 2.2. Computational Validation

The use of calculation models to predict the solubility of chemicals in mixed solvents is one of the lines of research that have evolved the most in recent years. Some of the most widely implemented models are those of, van’t Hoff, Jouyban–Acree–van’t Hoff and Buchowski–Ksiazczak λh.

Thus, the van’t Hoff equation (Equation (3)) presents a relationship between solubility (expressed in mole fraction) and temperature.

(3) $$x_{3}=e^{\left(A\;+\;\frac{B}{T}\right)}$$

*A* and *B* are parameters, which can be related to thermodynamic parameters such as dissolution enthalpy and dissolution entropy [37].

Jouyban and Acree developed a specific model for the prediction of the solubility of drugs in {ethanol (1) + water (2)} cosolvent mixtures at a specific temperature (Equation (4)) [38,39,40]:

(4) $$\ln x_{3,1+2}=x_{1}\ln x_{3,1}\;+\;x_{2}\ln x_{3,2}+\;x_{1}x_{2}\left[724.21T^{-1}\;+\;485.17\left(x_{1}\;-x_{2}\right)T^{-1}\;+\;194.41{\left(x_{1}-x_{2}\right)}^{2}T^{-1}\right]$$

Introducing the van’t Hoff model, the Jouyban–Acree would be left, with the advantage of being able to calculate solubility at various temperatures [41].

(5) $$\ln x_{3,1+2}=x_{1}\left(A_{1}+B_{1}T^{-1}\right)+x_{2}\left(A_{2}+B_{2}T^{-1}\right)+x_{1}x_{2}\left[724.21T^{-1}\;+\;485.17\left(x_{1}\;-x_{2}\right)T^{-1}\;+\;194.41{\left(x_{1}-x_{2}\right)}^{2}T^{-1}\right]$$

For this investigation, when calculating A and B coefficients by linear regression, the following equation is obtained:

(6) $$\ln x_{3,1+2}=x_{1}\left(-0.69\pm 0.22-2048T^{-1}\right)+x_{2}\left(8.06\pm 0.28-4101\pm 87T^{-1}\right)\;+x_{1}x_{2}\left[724.21T^{-1}+485.17\left(x_{1}-x_{2}\right)T^{-1}\;+194.41{\left(x_{1}-x_{2}\right)}^{2}T^{-1}\right]$$

The Buchowski–Ksiazczak *λh* equation, (Equation (8)), is another way to describe the solubility behavior:

(7) $$x_{3}=\frac{\lambda e^{\lambda hT_{f}^{-1}}}{\lambda e^{\lambda hT_{f}^{-1}}-e^{\lambda hT_{f}^{-1}}+e^{\lambda hT^{-1}}}$$

where *λ* and *h* are the two parameters of the Buchowski–Ksiazczak model, and *T_f_* represents the melting point of drug [42,43,44].

The mean percentage deviation (MPD) was calculated from Equation (8) [45,46]:

(8) $$\mathrm{MPD}=\frac{100}{N}\sum \frac{\left|x_{3,1+2}^{\mathrm{cal}}-x_{3,1+2}^{\mathrm{Exp}}\right|}{x_{3,1+2}^{\mathrm{Exp}}}$$

where *N* is the number of experimental data points, and ${\;x}_{3,1+2}^{\mathrm{cal}}$ and $x_{3,1+2}^{\mathrm{Exp}}$ are the calculated and experimental solubility values.

Thus, amygdalin solubility was estimated employing Equations (3), (6), and (7) and then, the MPD values were calculated using Equation (8).

The MDP values show that the model that best predicts the experimental data is the van’t Hoff model (3.3%), followed by the Buchowski–Ksiazczak model (4.3%), and finally, the Jouyban–Acree–van’t Hoff mode presents a MDP of 22.8%.

**Figure 3:**
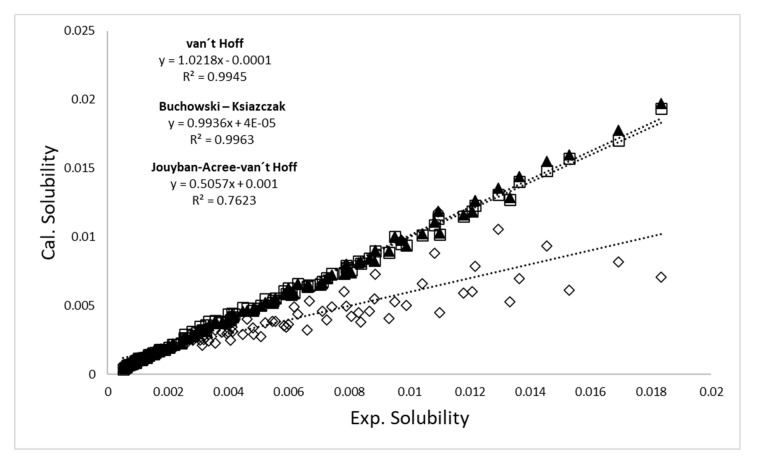
Plot of calculated versus experimental solubility data of amygdalin in {ethanol (1) + water (2)} cosolvent mixtures (▲ = van’t Hoff, □ = Buchowski–Ksiazczak, and ◊ = Jouyban–Acree–van’t Hoff).

Figure 3 shows the calculated solubility versus observed solubility data of amygdalin in {ethanol (1) + water (2)} cosolvent mixtures, using the van’t Hoff, Jouyban–Acree–van’t Hoff and Buchowski–Ksiazczak models. A relatively low determination coefficient was observed (*R*^2^ = 0.76) indicating a poor prediction accuracy for the Jouyban–Acree–van’t Hoff model; however, the van’t Hoff and Buchowski–Ksiazczak models present correlation coefficients close to one, indicating a good correlation of the data calculated with these models and the experimental data.

Therefore, in general terms, the Jouyban–Acree–van’t Hoff model does not predict the solubility of amygdalin in {ethanol (1) + water (2)} cosolvent mixtures adequately; however, the van’t Hoff and Buchowski–Ksiazczak models show very good precision, as demonstrated with the MDP values.

### 2.3. Thermodynamic Functions of Dissolution

From the experimental solubility data (Table 1), the thermodynamic functions of dissolution (Table 3) were calculated using the van’t Hoff and Gibbs equations, under Krug modifications [47,48]:

(9) $${∆}_{\mathrm{soln}}H^{o}=-R\left(\partial \ln x_{3}/\partial \left(T^{-1}-T_{\mathrm{hm}}^{-1}\right)\right)$$

(10) $${∆}_{\mathrm{soln}}G^{o}=-R\times T_{\mathrm{hm}}\times \mathrm{intercept}$$

(11) $${∆}_{\mathrm{soln}}S^{o}=\left({∆}_{\mathrm{soln}}H^{o}-{∆}_{\mathrm{soln}}G^{o}\right)T_{\mathrm{hm}}^{-1}$$

where ${∆}_{\mathrm{soln}}H^{o}$ represents the solution standard enthalpy, ${∆}_{\mathrm{soln}}S^{o}$ represents the solution standard entropy, ${∆}_{\mathrm{soln}}G^{o}$ represents the solution standard Gibbs energy, R represents the constant of gases, and *T*_hm_ represents the mean harmonic temperature defined as: *T*_hm_ = *n*/Σ(1/*T*), where *n* is the number of studied temperatures (the harmonic mean temperature for this investigation is 310.22 K).

Upon graphing ln x_3_ vs. (*T*^−1^ − *T*_hm_^−1^), the slope (∂ln x_3_/∂ (*T*^−1^ − *T*_hm_^−1^)) and intercept used in Equations (10) and (11) are obtained.

**Figure 4:**
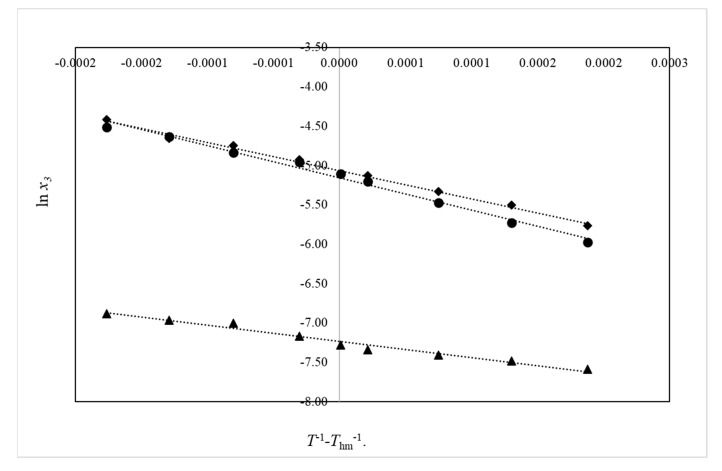
The van’t Hoff plots for amygdalin in {ethanol (1) + water (2)} cosolvent mixtures (●: x_1_ = 0.0; ♦: x_1_ = 0.5; and ▲: x_1_ = 1.0).

Table 3 shows the data for the apparent thermodynamic functions of solution for amygdalin, Δ_soln_*H^o^*, Δ_soln_*G^o^*,and Δ_soln_*S^o^*. The values of the slope and intercept with their respective standard deviations were calculated using the TableCurve 2D program. The resulting graphs are linear for each of the EtOH + W cosolvent mixtures, obtaining correlation coefficients very close to 1 for first order linear regressions (*y* = a + b*x*) (Figure 4).

The standard Gibbs energy Δ_soln_*G*° (Table 3) is positive over the whole composition range and decreases from neat water to the cosolvent mixture *x*_1_ = 0.4. From this solvent composition to pure EtOH, Δ_soln_*G*^°^ increases. The Δ_soln_*H*^°^ is positive in every case indicating that the process of dissolution of amygdalin powder in solvents is endothermic [49,50]. The enthalpic values increase nonlinearly from neat water up to 40% in volume of EtOH, presumably because, by increasing ethanol content, the interaction of this solvent with the solute promotes the breaking of the structured water molecules (hydrogen bonds) around the non-polar group of amygdalin [33,51]. As for the standard entropy of solution (Δ_soln_*S*^°^), it is negative for pure ethanol, while it is positive for water-rich mixtures, suggesting an overall entropy-driven process for the latter mixtures. The relative contributions by enthalpy (*ζ_H_*) and by entropy (*ζ_TS_)* toward standard Gibbs free energy of solution are given by Equations (12) and (13), respectively:

(12) $$\zeta_{H}=\left|\Delta_{\mathrm{soln}}H^{o}\right|{\left(\left|\Delta_{\mathrm{soln}}H^{o}\right|+\left|T_{\mathrm{hm}}\Delta_{\mathrm{soln}}S^{o}\right|\right)}^{-1}$$

(13) $$\zeta_{TS}=\left|T_{\mathrm{hm}}\Delta_{\mathrm{soln}}S^{o}\right|{\left(\left|\Delta_{\mathrm{soln}}H^{o}\right|+\left|T_{\mathrm{hm}}\Delta_{\mathrm{soln}}S^{o}\right|\right)}^{-1}$$

It may be seen from Table 4 that, in all cases, the main contributor to the (positive) standard Gibbs energy of dissolution is the (positive) enthalpy term (*ζ_H_* > 0.59).

### 2.4. Enthalpy–Entropy Compensation

The study of enthalpy–entropy compensation effects for solute dissolution has been used to identify the main mechanism involved in the cosolvent behavior on dissolution processes [52,53]. Plots of ∆_soln_*H^o^* as a function of ∆_soln_*G^o^* or T∆_soln_*S^o^* at the harmonic temperature are employed for this purpose.

Thus, when plotting ∆_soln_*H^o^* vs. ∆_soln_*G^o^*, a positive slope will indicate an enthalpy-driven dissolution process, while a negative one will indicate an entropy-driven dissolution process [39].

Similarly, when plotting ∆_soln_H^o^ vs. T∆_soln_S^o^, a slope greater than one will indicate an enthalpy-driven dissolution processes while a slope of less than one indicates entropy-driven dissolution processes [7,52,53,54,55,56].

**Figure 5:**
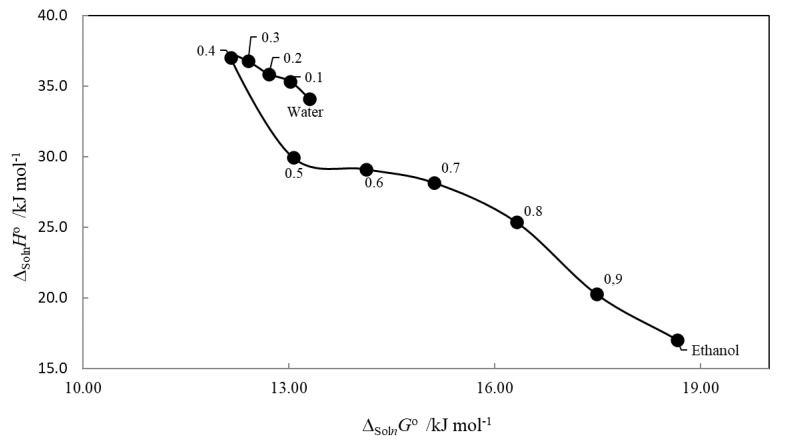
Enthalpy–entropy compensation graph of Δ_soln_*H*° vs. Δ_soln_*G*° at *T*_hm_ = 310.22 K.

Figure 5 shows that amygdalin in {ethanol (1) + water (2)} mixture solvents exhibits two trends, both with a negative slope, suggesting that the whole dissolution process is driven by entropy.

**Figure 6:**
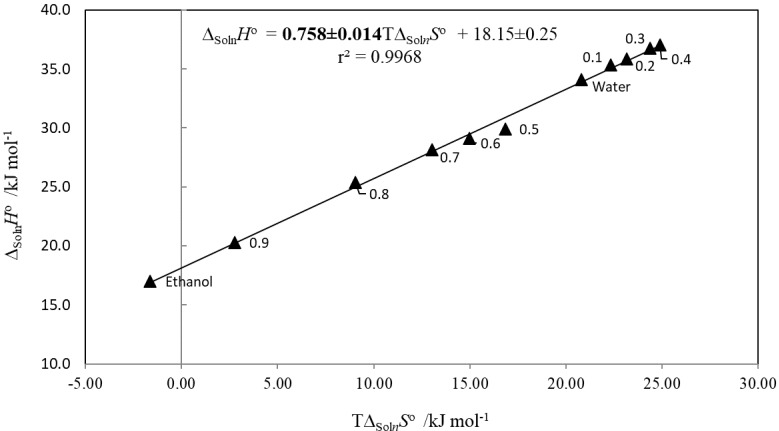
Enthalpy–entropy compensation graph of Δ_soln_*H_o_* vs. *T*Δ_soln_*S_o_* at *T*_hm_ = 310.22 K.

When plotting ∆_soln_*H*^o^ vs. *T*∆_soln_*S*^o^ (Figure 6), a linear trend is observed, described by the following equation: ∆_Soln_*H*^o^ = 0.758 ± 0.014*T*∆_Soln_*S*^o^ + 18.15 ± 0.25. The slope is inferior to 1, which corroborates the previous results.

### 2.5. Preferential Solvation

The preferential solvation model suggested by Ben Naim, called the inverse Kirkwood–Buff Integral (IKBI), allows determining, at the molecular level, the arrangement of the solvent molecules that make up the cosolvent mixture around a dissolved solute molecule [57,58,59].

This model allows to obtain the preferential solvation parameter of amygdalin (3) by ethanol molecules ($\delta x_{1,3})$ according to [60,61,62]:

(14) $$\delta x_{1,3}=\left[x_{1}\left(1-x_{1}\right)\left(G_{1,3}-G_{2,3}\right)\right]{\left[x_{1}G_{1,3}+\left(1-x_{1}\right)G_{2,3}+V_{cor}\right]}^{-1}$$

where *x*_1_ is the molar fraction of ethanol-free amygdalin, *G*_1,3_ and *G*_2,3_ are the Kirkwood–Buff integrals (cm^3^ mol^−1^), and *V_cor_* is the correlation volume (cm^3^ mol^−1^).

Thus, $G_{1,3}$ and $G_{2,3}$ are calculated as [63,64]:

(15) $$G_{1,3}=RT\kappa_{T}-V_{3}+(1-x_{1})V_{2}DQ^{-1}$$

(16) $$G_{2,3}=RT\kappa_{T}-V_{3}+x_{1}V_{1}DQ^{-1}$$

where *κ_T_* is the isothermal compressibility of ethanol + water mixtures (GPa^−1^), *V*_1_ and *V*_2_ are the molar volumes of ethanol and water, respectively, in the mixtures (cm^3^ mol^−1^), and *V*_3_ is the molar volume of amygdalin in the mixed solvent (cm^3^ mol^−1^)

*V_cor_* is defined as [65,66]:

(17) $$V_{cor}=2522.5{\left[r_{3}+0.1363\sqrt[{3}]{x_{1,3}^{L}V_{1}+x_{2,3}^{L}V_{2}}-0.085\right]}^{3}$$

where *x*_1,3_^*L*^ is the local molar fraction of ethanol (1) in the surrounding area of amygdalin (3) and *r*_3_ is the amygdalin molecular radius (nm).

*V_cor_* is calculated by iteration using Equations (15) and (18) [67]:

(18) $$\;\;\;\;\;\;\;\;\delta x_{1,3}=x_{1,3}^{L}-x_{1}$$

The functions *D* and *Q* (kJ mol^−1^) are calculated using the following equations:

(19) $$Q=RT+x_{1}x_{2}{\left(\frac{\partial^{2}G_{1,2}^{E}}{\partial x_{2}^{2}}\right)}_{T,P}$$

(20) $$D={\left(\partial {∆}_{tr}G_{3,2\to 1+2}^{o}/\partial x_{1}\right)}_{T,P}$$

∆*_tr_G*^o^_3,2→1 + 2_ is the standard molar Gibbs energy of transfer of the solute from pure water to each {ethanol (1) + water (2)} mixture and *G^E^*_1,2_ is the excess molar Gibbs energy of mixing of the two solvents free of amygdalin.

**Figure 7:**
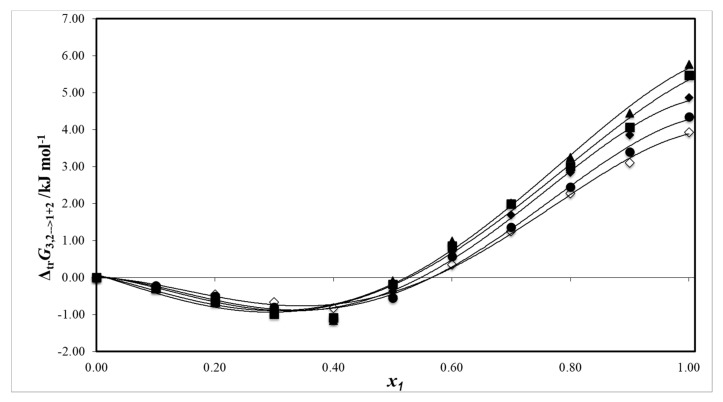
Gibbs energy of transfer of amygdalin (3) from pure water (2) to {ethanol (1) + water (2)} mixtures at several temperatures: □: 293.15 K; ●: 298.15 K; ♦: 303.15 K; ■: 308.15 K; and ▲: 313.15 K.

Figure 7 shows the behavior of the Gibbs energy of transfer of amygdalin (3) from pure water (2) to {ethanol (1) + water (2)} mixtures at several temperatures. The numerical values were computed from the experimental solubility data (Table 1), by using the following equation:

(21) $${∆}_{tr}G_{3,2\to 1+2}^{o}=RT\ln\left(x_{3,2}x_{3,1+2}^{-1}\right)$$

(22) $${∆}_{tr}G_{3,2\to 1+2}^{o}=a+bx_{1}+cx_{1}{}^{2}+dx_{1}{}^{3}+ex_{1}{}^{4}$$

Table 4 records the numerical values of the coefficients of Equation (22) at 293.15, 298.15, 303.15, 308.15, and 313.15 K.

On the other hand, Q is calculated according to Equation (19), where *G*_1.2_^E^ is calculated as [64]:

(23) $$G_{1,2}^{E}=x_{1}x_{2}\left[2907-777\left(1-2x_{1}\right)+494{\left(1-2x_{2}\right)}^{2}\right]$$

Once D and Q are calculated together with the isothermal compressibility (κ_T_) for water (0.457 GPa^−1^) [68] and ethanol (1.248 GPa^−1^), in addition to the molar volumes of amygdalin and the solvents in the binary mixture reported by Jiménez et al. [69], the Kirkwood–Buff integrals are calculated, and from these, the preferential solvation parameters δ*x*_1,3_ of amygdalin in the binary solvent mixtures at the studied temperatures are calculated [70].

According to the literature, positive values of δ*x*_1,3_ indicate preferential solvation of amygdalin by ethanol. Conversely, negative values of δ*x*_1,3_ indicate preferential solvation of amygdalin by water.

**Figure 8:**
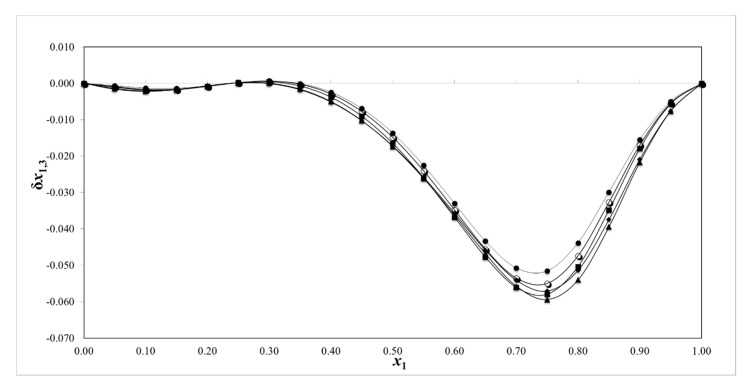
The *δx*_1,3_ values of amygdalin (3) in {ethanol (1) + water (2)} mixtures at mixtures ●: 293.15 K; ○: 298.15 K; ♦: 303.15; ■: 308.15 K; and ▲: 313.15 K.

The values of *δx*_1,3_ are presented in Table 5 and the behavior of *δx*_1,3_ is illustrated in Figure 8. Thus, from neat water to x_1_ = 0.45, the absolute value of *δx*_1,3_ is inferior to 0.01, indicating insignificant preferential solvation, probably because the values are within the error of the measurement [60]. From this composition to pure ethanol, the values of *δx*_1,3_ are negative and greater than 0.01. The maximum negative *δx*_1,3_ value is reached in the mixture x_1_ = 0.75 (Figure 8). These results indicate the preferential solvation of amygdalin by water. Because of the availability of two sugar moieties in the molecular structure of amygdalin, this molecule can form hydrogen bonds with proton-acceptor solvents. At the same time, amygdalin can act as a proton-acceptor (base group) molecule due to the free electron pair of the oxygen atom in the OH group or nitrogen atom of the C≡N group. Thus, the tendency of amygdalin for water in ethanol-rich mixtures could be explained in the matter of the greater acidic character of water (1.17 for water and 0.86 for ethanol, as stated in the acid scale of Taft and Kamlet [71]) interacting with proton-acceptor groups of amygdalin.

## 3. Experimental procedures

### 3.1. Reagents

Amygdalin (purity 98%) and HPLC-grade ethanol (purity 99.9%) were acquired from Sigma-Aldrich (San Luis, WA, USA).

Doubly distilled and deionized water were used in all experiments. All chemicals were used without further purification.

### 3.2. Solubility Determination

The employed techniques to prepare ethanol–water binary solvent mixtures and to measure the solubility of amygdalin in these solvents were used as reported in different studies [72,73,74]. The solubility of amygdalin in pure and mixed solvents was investigated at different temperatures in the range of 298.15–328.15 K. The gravimetric method was used to measure the composition of the saturated solutions.

The solvent mixtures were prepared by mass using a Sartorius balance (CP225D) with an accuracy of ±0.01 mg. An excess of amygdalin powder was added to the liquid phase, and the saturated solutions were brought into a twofold jacketed reactor (Polystat Huber CC2) at T ± 0.1 K. The solutions are magnetically stirred at the desired temperature for at least 72 h to ensure the saturation equilibrium. Thereafter, they were allowed to settle for 2 h before sampling.

The supernatant solutions were withdrawn, filtered through a 0.45-µm syringe filter, and then dried in a vacuum oven at 328.15 K. The mass of the dried samples was periodically measured using an analytical balance until stability. All determinations were performed three times to check reproducibility, and then an average value was taken to determine the amygdalin solubility in all systems at each condition. The solubility of amygdalin was calculated by molar fraction ($x_{A}$) in pure and different binary ethanol–water mixtures using the Equations (1) and (2), respectively.

## 4. Conclusions

The solubilities of amygdalin in {ethanol (1) + water (2)} mixtures were determined at different temperatures. The maximum solubility was obtained in 0.4-mole fraction of ethanol at 328.15 K and the lowest one in pure ethanol at 293.15 K. The amygdalin solubility was calculated using the van’t Hoff, Jouyban–Acree-van’t Hoff, and Buchowski-–Ksiazczak models, the data obtained using the Jouyban–Acree–van’t Hoff model showing important deviations with respect to experimental solubility; however, the van’t Hoff and Buchowski–Ksiazczak models showed a good correlation with the experimental data. As for solution thermodynamics, an endothermic process was observed, with a pronounced enthalpic contribution, but with entropic conduction.

The IKBI approach demonstrated that amygdalin is preferentially solvated by water in ethanol-rich mixtures, which is consistent with the decrease in amygdalin solubility by the addition of ethanol. Whereas, in water-rich mixtures (0 < *x*_1_ < 0.45), the solvent that will solvate the amygdalin molecule was not well defined.

In general terms, the data presented in this research expand the physicochemical information of amygdalin in binary aqueous-cosolvent mixtures, which are very useful, both for the pharmaceutical industry and for research processes related to this drug.

## Figures and Tables

**Table 1:** Experimental solubility of amygdalin (3) expressed in molar fraction (10^3^x_3_^a^) in [ethanol (1) + water (2)] mixtures at different temperatures. Experimental pressure p: 0.1 MPa ^b^.

*x*_1_^c,d^	Temperature/K ^e^								
	293.15	298.15	303.15	308.15	310.15	313.15	318.15	323.15	328.15
0.00	2.54	3.25	4.19	5.49	6.07	7.04	7.91	9.73	11.0
0.10	2.80	3.55	4.61	6.16	6.69	7.83	8.86	10.8	12.9
0.20	3.07	3.99	5.21	7.12	7.42	8.84	10.4	12.2	14.6
0.30	3.34	4.48	5.84	8.08	8.32	9.90	11.8	13.6	16.9
0.40	3.56	5.06	6.60	8.39	9.32	11.0	13.3	15.3	18.3
0.50	3.12	4.06	4.84	5.90	5.99	7.26	8.67	9.52	12.1
0.60	2.20	2.58	3.17	3.94	4.14	4.81	5.55	6.30	7.89
0.70	1.53	1.88	2.14	2.52	2.83	3.25	3.77	4.12	5.45
0.80	1.00	1.21	1.36	1.70	1.76	2.02	2.44	2.62	2.93
0.90	0.71	0.83	0.91	1.12	1.15	1.27	1.35	1.57	1.70
1.00	0.51	0.56	0.61	0.65	0.69	0.77	0.91	0.94	1.03

^a^ Average relative uncertainty in mole fraction solubility is u(x_3_) = 0.025. ^b^ Standard uncertainty in pressure u(p) = 0.001 MPa. ^c^ *x*_1_ is the mole fraction of ethanol (1) in the {ethanol (1) + water (2)} mixtures free of amygdalin (3). ^d^ Average relative standard uncertainty in *x*_1_ is ur(x_1_) = 0.01. ^e^ *T* is the absolute temperature. Standard uncertainty in temperature is u(T) = 0.05 K.

**Table 2:** Estimation of the solubility parameter of amygdalin by Fedor’s method [34].

Group or Atom	Quantity	∆V (cm^3^ mol^−1^)	∆U (kJ mol^−1^)
–CH_2_	2	16.1	4.94
–CH<	11	13.5	4.31
–OH	7	10	29.8
–O–	4	3.8	3.35
–C≡N	1	24	25.5
Phenyl	1	71.4	31.9
Ring closure	1	16	1.05
Total		377.3	337.74
			*δ*_3_ = (337,740/377.3)^1/2^ = 29.9 MPa^1/2^

**Table 3:** Thermodynamic functions of dissolution processes of amygdalin in {ethanol (1) + water (2)} cosolvent mixtures at *T*_hm_ = 310.22 K.

*x*_1_^a^	Δ_soln_*G^o^*/ kJ mol^−1^	Δ_soln_*H^o^*/ kJ mol^−1^	Δ_soln_*S^o^*/ J mol^−1^ K^−1^	TΔ_soln_*S^o^*/ kJ mol^−1^	ζ_H_^b^	ζ_TS_^b^
0.00	13.31	34.10	67.03	20.79	0.621	0.379
0.10	13.02	35.34	71.93	22.31	0.613	0.387
0.20	12.71	35.86	74.62	23.15	0.608	0.392
0.30	12.41	36.77	78.52	24.36	0.601	0.399
0.40	12.15	37.04	80.21	24.88	0.598	0.402
0.50	13.07	29.92	54.31	16.85	0.640	0.360
0.60	14.13	29.10	48.26	14.97	0.660	0.340
0.70	15.12	28.14	41.95	13.01	0.684	0.316
0.80	16.32	25.36	29.14	9.04	0.737	0.263
0.90	17.48	20.26	8.96	2.78	0.879	0.121
1.00	18.66	17.03	−5.25	−1.63	0.913	0.087

^a^*x*_1_ is the mole fraction of ethanol (1) in the {ethanol (1) + water (2)} mixtures free of amygdalin (3). Standard uncertainty in *T* is *u*(*T*) = 0.10 K. Average relative standard uncertainties in apparent thermodynamic quantities of real dissolution processes are *u*_r_(∆_soln_*G*°) = 0.02, *u*_r_(∆_soln_*H*°) = 0.02, *u*_r_(∆_soln_*S*°) = 0.03, and *u*_r_(*T*∆_soln_*S*°) = 0.03. ^b^ *ζ_H_* and *ζ_TS_* are the relative contributions by enthalpy and entropy toward apparent Gibbs energy of dissolution.

**Table 4:** Coefficients of the Equation (22) to Gibbs energy of transfer of amygdalin (3) at several temperatures.

Coefficient	293.15 K	298.15 K	303.15 K	308.15 K	313.15 K
*a*	−0.0022	0.0356	0.0446	0.0879	0.0706
*b*	−0.3574	−1.2782	1.8586	−5.1881	−3.8227
*c*	−20.48	−19.625	−18.477	−1.9751	−8.7567
*d*	53.775	55.874	57.349	32.26	43.505
*e*	−29.053	−30.735	32.277	−19.838	−25.332
*R* ^2^	0.998	0.994	0.995	0.993	0.994

**Table 5:** The *δx*_1,3_ values of amygdalin in {ethanol (1) + water (2)} mixtures at some temperatures.

*x*_1_^ a^	*δx_1,3_*				
	293.15	298.15	303.15	308.15	313.15
0.000	0.000	0.000	0.000	0.000	0.000
0.050	−0.001	−0.001	−0.001	−0.002	−0.001
0.100	−0.001	−0.002	−0.002	−0.002	−0.002
0.150	−0.001	−0.002	−0.002	−0.002	−0.002
0.200	−0.001	−0.001	−0.001	−0.001	−0.001
0.250	0.000	0.000	0.000	0.000	0.000
0.300	0.001	0.001	0.000	0.000	0.000
0.350	0.000	0.000	−0.001	−0.002	−0.002
0.400	−0.002	−0.003	−0.004	−0.005	−0.005
0.450	−0.007	−0.008	−0.009	−0.010	−0.010
0.500	−0.014	−0.015	−0.017	−0.017	−0.017
0.550	−0.023	−0.024	−0.026	−0.026	−0.026
0.600	−0.033	−0.035	−0.037	−0.036	−0.036
0.650	−0.043	−0.046	−0.048	−0.046	−0.047
0.700	−0.051	−0.054	−0.056	−0.054	−0.056
0.750	−0.052	−0.055	−0.058	−0.057	−0.059
0.800	−0.044	−0.047	−0.050	−0.051	−0.054
0.850	−0.030	−0.033	−0.035	−0.037	−0.039
0.900	−0.016	−0.017	−0.018	−0.021	−0.022
0.950	−0.005	−0.005	−0.006	−0.008	−0.008
1.000	0.000	0.000	0.000	0.000	0.000

^a^*x*_1_ is the mole fraction of ethanol (1) in the {ethanol (1) + water (2)} mixtures free of amygdalin (3).

## References

[1] Jaswal V., Palanivelu J., Ramalingam C. (2018). Effects of the Gut microbiota on Amygdalin and its use as an anti-cancer therapy: Substantial review on the key components involved in altering dose efficacy and toxicity. Biochem. Biophys. Rep..

[2] Guo J., Wu W., Sheng M., Yang S., Tan J. (2013). Amygdalin inhibits renal fibrosis in chronic kidney disease. Mol. Med. Rep..

[3] Carter J.H., McLafferty M.A., Goldman P. (1980). Role of the gastrointestinal microflora in Amygdalin (laetrile)-induced cyanide toxicity. Biochem. Pharmacol..

[4] Li Y., Li Q., Liu R., Shen X. (2015). Chinese Medicine Amygdalin and β-Glucosidase Combined with Antibody Enzymatic Prodrug System as A Feasible Antitumor Therapy. Chin. J. Integr. Med..

[5] Moon J.Y., Kim S.W., Yun G.M., Lee H.S., Kim Y.D., Jeong G.J., Ullah I., Rho G.J., Jeon B.G. (2015). Inhibition of cell growth and down-regulation of telomerase activity by Amygdalin in human cancer cell lines. Anim. Cells Syst..

[6] Song Z., Xu X. (2014). Advanced research on anti-tumor effects of Amygdalin. J. Cancer Res. Ther..

[7] Zhang X., Hu J., Zhuo Y., Cui L., Li C., Cui N., Zhang S. (2018). Amygdalin improves microcirculatory disturbance and attenuates pancreatic fibrosis by regulating the expression of endothelin-1 and calcitonin gene-related peptide in rats. J. Chin. Med. Assoc..

[8] Hwang H.J., Lee H.J., Kim C.J., Shim I., Hahm D.H. (2008). Inhibitory effect of Amygdalin on lipopolysaccharide-inducible TNF-alpha and IL-1beta mRNA expression and carrageenan-induced rat arthritis. J. Microbiol. Biotechnol..

[9] Zhu Y.P., Su Z.W., Li C.H. (1994). Analgesic effect and no physical dependence of Amygdalin. China J. Chin. Mater. Med..

[10] Jiagang D., Li C., Wang H., Hao E., Du Z., Bao C., Lv J., Wang Y. (2011). Amygdalin mediates relieved atherosclerosis in apolipoprotein E deficient mice through the induction of regulatory T cells. Biochem. Biophys. Res. Commun..

[11] Zhao F., Yang Z. (2012). Amygdalin attenuates atherosclerosis progress through inhibiting of toll-like receptors expression and activity. J. Anim. Vet. Adv..

[12] Wang Z., Fang K., Wang G., Guan X., Pang Z., Guo Y., Yuan Y., Ran N., Liu Y., Wang F. (2019). Protective effect of Amygdalin on epithelial—mesenchymal transformation in experimental chronic obstructive pulmonary disease mice. Phytother. Res..

[13] He X.Y., Wu L.J., Wang W.X., Xie P.J., Chen Y.H., Wang F. (2020). Amygdalin-A pharmacological and toxicological review. J. Ethnopharmacol..

[14] Nabavizadeh F., Alizadeh A.M., Sadroleslami Z., Adeli S. (2011). Gastroprotective effects of Amygdalin on experimental gastric ulcer: Role of NO and TNF-α. J. Med. Plant Res..

[15] Lee H.M., Moon A. (2016). Amygdalin regulates apoptosis and adhesion in Hs578T triple-negative breast cancer cells. Biomol. Ther..

[16] Haisman D.R., Knight D.J. (1967). The enzymic hydrolysis of Amygdalin. Biochem. J..

[17] Bolarinwa I.F., Orfila C., Morgan M.R. (2014). Amygdalin content of seeds, kernels and food products commercially-available in the UK. Food Chem..

[18] Krieble V.K. (1912). The amygdalins and their inter-reactions with emulsin. J. Am. Chem. Soc..

[19] Boháčová I., Procházková S., Halko R. (2019). Separation and determination of Amygdalin and unnatural neoAmygdalin in natural food supplements by HPLC-DAD. Food Addit. Contam. Part A.

[20] Thapa R.K., Choi H.G., Kim J.O., Yong C.S. (2017). Analysis and optimization of drug solubility to improve pharmacokinetics. J. Pharm. Investig..

[21] Pacheco D.P., Martínez F. (2007). Thermodynamic analysis of the solubility of naproxen in ethanol + water cosolvent mixtures. Phys. Chem. Liq..

[22] Di L., Fish P.V., Mano T. (2012). Bridging solubility between drug discovery and development. Drug Discov. Today.

[23] Sun H., Li M., Jia J., Tang F., Duan E. (2012). Measurement and correlation of the solubility of 2, 6-diaminohexanoic acid hydrochloride in aqueous methanol and aqueous ethanol mixtures. J. Chem. Eng. Data..

[24] Delgado D.R., Martínez F. (2013). Solubility and solution thermodynamics of sulfamerazine and sulfamethazine in some ethanol+ water mixtures. Fluid Phase Equilibria.

[25] Pobudkowska A., Domańska U., Jurkowska B.A., Dymczuk K. (2015). Solubility of pharmaceuticals in water and alcohols. Fluid Phase Equilibria.

[26] Allen L., Ansel H.C. (2013). Ansel’s Pharmaceutical Dosage Forms and Drug Delivery Systems.

[27] Wijesekera R.O.B. (2017). The Medicinal Plant Industry.

[28] Marcus Y. (2013). Preferential solvation in mixed solvents. Fluctuation Theory of Solutions: Applications in Chemistry, Chemical Engineering, and Biophysics.

[29] Marcus Y. (2017). Preferential solvation of drugs in binary solvent mixtures. Pharm. Anal. Acta.

[30] Yalkowsky S.H. (1999). Solubility and Solubilization in Aqueous Media.

[31] Williams R.O., Watts A.B., Miller D.A. (2016). Formulating Poorly Water Soluble Drugs.

[32] Delgado D.R., Rodríguez G.A., Martínez F. (2013). Thermodynamic study of the solubility of sulfapyridine in some ethanol + water mixtures. J. Mol. Liq..

[33] Muñoz M.d., Delgado D.R., Peña M.Á., Jouyban A., Martínez F. (2015). Solubility and preferential solvation of sulfadiazine, sulfamerazine and sulfamethazine in propylene glycol + water mixtures at 298.15 K. J. Mol. Liq..

[34] Barton A. (1991). CRC Handbook of Solubility Parameters and Other Cohesion Parameters.

[35] Delgado D.R., Mogollon-Waltero E.M., Ortiz C.P., Peña M., Almanza O.A., Martínez F., Jouyban A. (2018). Enthalpy-entropy compensation analysis of the triclocarban dissolution process in some {1,4-dioxane (1) + water (2)} mixtures. J. Mol. Liq..

[36] Hildebtand J.H., Prausnitz J.M., Scott R.L. (1970). Regular and Related Solutions: The Solubility of Gases, Liquids, and Solids.

[37] Grant D.J.W., Mehdizadeh M., Chow A.H.L., Fairbrother J.E. (1984). Non-linear van’t Hoff solubility-temperature plots and their pharmaceutical interpretation. Int. J. Pharm..

[38] Jouyban A., Acree W.E. (2018). Mathematical derivation of the Jouyban-Acree model to represent solute solubility data in mixed solvents at various temperatures. J. Mol. Liq..

[39] Ruidiaz M., Delgado D.R., Martínez F. (2010). Correlating the solubility of indomethacin in 1,4-dioxane + water mixtures by means of the Jouyban-Acree model. Rev. Colomb. Cienc. Químico Farm..

[40] Acree W., Jouyban A., Acree W.E. (2006). In silico prediction of drug solubility in water-ethanol mixtures using Jouyban-Acree model. J. Pharm Pharm. Sci..

[41] Nieto A.M.R., Cerquera N.E., Delgado D.R. (2019). Measurement and correlation of solubility of ethylparaben in pure and binary solvents and thermodynamic properties of solution. Rev. Colomb. Cienc. Químico Farm..

[42] Ksia̧ẑczak A., Kosinski J.J. (1988). Vapour pressure of binary, three-phase (S-L-V) systems and solubility. Fluid Phase Equilibria.

[43] Ksiazczak A., Moorthi K., Nagata I. (1994). Solid-solid transition and solubility of even n-alkanes. Fluid Phase Equilibria.

[44] Krajangsod S., Chotikamas S., Tawai A., Sriariyanun M. (2018). Measurement and thermodynamic modelling of erythritol solubility in aqueous solvents. Orient. J. Chem..

[45] Blanco-Márquez J.H., Quigua-Medina Y.A., García-Murillo J.D., Castro-Camacho J.K., Ortiz C.P., Cerquera N.E., Delgado D.R. (2020). Thermodynamic analysis and applications of the Abraham solvation parameter model in the study of the solubility of some sulfonamides. Rev. Colomb. Cienc. Químico-Farm..

[46] Khoubnasabjafari M., Delgado D.R., Martinez F., Jouyban A., Acree W.E. (2020). Predicting the solubility, thermodynamic properties and preferential solvation of sulphamethazine in {acetonitrile + water} mixtures using a minimum number of experimental data points. Phys. Chem. Liq..

[47] Krug R.R., Hunter W.G., Grieger R.A. (1976). Enthalpy-entropy compensation. 1. Some fundamental statistical problems associated with the analysis of van’t Hoff and arrhenius data. J. Phys. Chem..

[48] Krug R.R., Hunter W.G., Grieger R.A. (1976). Enthalpy-entropy compensation. 2. Separation of the chemical from the statistical effect. J. Phys. Chem..

[49] Delgado D.R., Jouyban A., Martínez F. (2014). Solubility and preferential solvation of meloxicam in methanol+ water mixtures at 298.15 K. J. Mol. Liq..

[50] Blanco-Márquez J.H., Ortiz C.P., Cerquera N.E., Martínez F., Jouyban A., Delgado D.R. (2019). Thermodynamic analysis of the solubility and preferential solvation of sulfamerazine in (acetonitrile + water) cosolvent mixtures at different temperatures. J. Mol. Liq..

[51] Valvani S.C., Yalkowsky S.H., Amidon G.L. (1976). Solubility of nonelectrolytes in polar solvents. VI. Refinements in molecular surface area computations. J. Phys. Chem..

[52] Machatha S.G., Bustamante P., Yalkowsky S.H. (2004). Deviation from linearity of drug solubility in ethanol/water mixtures. Int. J. Pharm..

[53] Volkova T.V., Levshin I.B., Perlovich G.L. (2020). New antifungal compound: Solubility thermodynamics and partitioning processes in biologically relevant solvents. J. Mol. Liq..

[54] Perlovich G.L., Strakhova N.N., Kazachenko V.P., Volkova T.V., Tkachev V.V., Schaper K.J., Raevsky O.A. (2008). Sulfonamides as a subject to study molecular interactions in crystals and solutions: Sublimation, solubility, solvation, distribution and crystal structure. Int. J. Pharm..

[55] Bustamante P., Escalera B. (1995). Enthalpy and Entropy Contributions to the Solubility of Sulphamethoxypyridazine in Solvent Mixtures Showing Two Solubility Maxima. J. Pharm. Pharmacol..

[56] Bustamante E.P., Ochoa R., Reillo A., Escalera J.-B. (1994). Chameleonic Effect of Sulfanilamide and Sulfamethazine in Solvent Mixtures. Solubility Curves with Two Maxima. Chem. Pharm. Bull..

[57] Ben-Naim A. (1988). Theory of preferential solvation of nonelectrolytes. Cell Biophys..

[58] Ben-Naim A. (2013). Theoretical aspects of self-assembly of proteins: A Kirkwood-Buff-theory approach. J. Chem. Phys..

[59] Ben-Naim A., Navarro A.M., Leal J.M. (2008). A Kirkwood-Buff analysis of local properties of solutions. Phys. Chem. Chem. Phys..

[60] Marcus Y. (2002). Solvent Mixtures: Properties and Selective Solvation.

[61] Marcus Y. (2008). On the preferential solvation of drugs and PAHs in binary solvent mixtures. J. Mol. Liq..

[62] Delgado D.R., Vargas E.F., Martínez F. (2013). Preferential solvation of xylitol in ethanol + water co-solvent mixtures according to the IKBI and QLQC methods. Rev. Colomb. Quim..

[63] Marcus Y. (2020). Preferential solvation in mixed solvents. 16. Mixtures of N,N-dimethylformamide or propylene carbonate with organic solvents. J. Chem. Thermodyn..

[64] Delgado D.R., Peña M.A., Martínez F. (2013). Preferential solvation of acetaminophen in ethanol + water solvent mixtures according to the inverse Kirkwood-Buff integrals method. Rev. Colomb Cienc. Químico Farm..

[65] Marcus Y. (2019). Preferential solvation in mixed solvents. 15. Mixtures of acetonitrile with organic solvents. J. Chem. Thermodyn..

[66] Castro J.J.S., Ortiz C.P., Rodríguez-Rubiano J.D., Rodríguez-Rodríguez G.A., Delgado D.R. (2018). Preferential solvation of tricin in {ethanol (1) + water (2)} mixtures at several temperatures. Rev. Colomb. Cienc. Químico Farm..

[67] Marcus Y. (1989). Preferential solvation. Part 3.—Binary solvent mixtures. J. Chem. Soc. Faraday Trans. 1 Phys. Chem. Condens. Phases..

[68] Marcus Y. (1999). The Properties of Solvents.

[69] Jiménez J., Manrique J., Martínez F. (2004). Effect of temperature on some volumetric properties for ethanol + water mixtures. Rev. Colomb. Cienc. Químico Farm..

[70] Aldana G.A.d.A., Rubio D.I.C., Rodríguez G.A.R., Lozano A.C., Mehrdad A., Delgado D.R., Carmona N.A.P. (2016). Solution thermodynamics and preferential solvation of 3-chloro-N-phenyl-phthalimide in acetone + methanol mixtures. Rev. Colomb. Cienc. Químico Farm..

[71] Taft R.W., Kamlet M.J. (1976). The Solvatochromic Comparison Method. 2. The α-Scale of Solvent Hydrogen-Bond Donor (HBD) Acidities. J. Am. Chem. Soc..

[72] Noubigh A. (2019). Stearic acid solubility in mixed solvents of (water + ethanol) and (ethanol + ethyl acetate): Experimental data and comparison among different thermodynamic models. J. Mol. Liq..

[73] Noubigh A., Aydi A., Abderrabba M. (2017). Experimental measurement and correlation of solubility data and thermodynamic properties of protocatechuic acid in four organic solvents. J. Chem. Eng. Data.

[74] Noubigh A., Akremi A. (2019). Solution thermodynamics of *trans*-Cinnamic acid in (methanol + water) and (ethanol + water) mixtures at different temperatures. J. Mol. Liq..

